# Evaluation of the Effects of Library Preparation Procedure and Sample Characteristics on the Accuracy of Metagenomic Profiles

**DOI:** 10.1128/mSystems.00440-21

**Published:** 2021-10-12

**Authors:** Christopher A. Gaulke, Emily R. Schmeltzer, Mark Dasenko, Brett M. Tyler, Rebecca Vega Thurber, Thomas J. Sharpton

**Affiliations:** a Department of Microbiology, Oregon State University, Corvallis, Oregon, USA; b Department of Pathobiology, University of Illinois at Urbana-Champaign, Urbana, Illinois, USA; c Carl R. Woese Institute for Genomic Biology, University of Illinois at Urbana-Champaign, Urbana, Illinois, USA; d Center for Quantitative Life Sciences, Oregon State University, Corvallis, Oregon, USA; e Department of Botany and Plant Pathology, Oregon State University, Corvallis, Oregon, USA; f Department of Statistics, Oregon State University, Corvallis, Oregon, USA; Duke University

**Keywords:** coral, gut, library preparation, metagenomics, microbiome, next-generation sequencing, soil

## Abstract

Shotgun metagenomic sequencing has transformed our understanding of microbial community ecology. However, preparing metagenomic libraries for high-throughput DNA sequencing remains a costly, labor-intensive, and time-consuming procedure, which in turn limits the utility of metagenomes. Several library preparation procedures have recently been developed to offset these costs, but it is unclear how these newer procedures compare to current standards in the field. In particular, it is not clear if all such procedures perform equally well across different types of microbial communities or if features of the biological samples being processed (e.g., DNA amount) impact the accuracy of the approach. To address these questions, we assessed how five different shotgun DNA sequence library preparation methods, including the commonly used Nextera Flex kit, perform when applied to metagenomic DNA. We measured each method’s ability to produce metagenomic data that accurately represent the underlying taxonomic and genetic diversity of the community. We performed these analyses across a range of microbial community types (e.g., soil, coral associated, and mouse gut associated) and input DNA amounts. We find that the type of community and amount of input DNA influence each method’s performance, indicating that careful consideration may be needed when selecting between methods, especially for low-complexity communities. However, the cost-effective preparation methods that we assessed are generally comparable to the current gold-standard Nextera DNA Flex kit for high-complexity communities. Overall, the results from this analysis will help expand and even facilitate access to metagenomic approaches in future studies.

**IMPORTANCE** Metagenomic library preparation methods and sequencing technologies continue to advance rapidly, allowing researchers to characterize microbial communities in previously underexplored environmental samples and systems. However, widely accepted standardized library preparation methods can be cost-prohibitive. Newly available approaches may be less expensive, but their efficacy in comparison to standardized methods remains unknown. In this study, we compared five different metagenomic library preparation methods. We evaluated each method across a range of microbial communities varying in complexity and quantity of input DNA. Our findings demonstrate the importance of considering sample properties, including community type, composition, and DNA amount, when choosing the most appropriate metagenomic library preparation method.

## INTRODUCTION

Recent advancements in high-throughput sequencing have revolutionized genomic discovery and unlocked new insights regarding the diversity and function of microbial communities (1–4). For example, shotgun metagenomic sequencing has clarified how the functional capacity of the gut microbiome links to human health (5–8), improved the efficacy of antibiotic resistance gene discovery (9–12), identified beneficial soil microbes for agricultural use (13–15), and uncovered novel, medically relevant biosynthetic gene clusters in marine microbes (16–18). However, while metagenomes offer rich opportunities to transform discovery, the financial cost of producing metagenomic data limits their application. Because much of this cost is associated with the preparation of metagenomic DNA for high-throughput sequencing, there is hope that emergent economical products and procedures can expand the scope of metagenomic investigations.

Illumina’s Nextera XT and DNA Flex kits (the latter now known as “Illumina DNA Prep”) have been the most widely used methods for preparing metagenomic libraries and have effectively served as industry-standard approaches. Indeed, Illumina DNA sequencing platforms remain the most widely utilized for generating genomic and metagenomic data, and their library preparation kits are accordingly used to prepare samples for sequencing. Due to their frequent use, these kits are subject to extensive evaluation and refinement. For example, Illumina recently released an updated version of their “gold-standard” Nextera XT kit, which was rebranded as Nextera DNA Flex (and now Illumina DNA Prep). This new kit allows greater flexibility across a wider range of genomes, from small genomes (microbial and amplicons) to more complex genomes found in eukaryotic and human systems. The Flex kit also resolved sequencing biases identified in the Nextera XT kit that occur in genomic regions with extreme GC content (19, 20). These features of the Nextera DNA Flex kit have contributed to its broad adoption in metagenomic investigations.

One downside to the Nextera DNA Flex kit is its relatively high price, which presently costs roughly $46 per sample. While this cost may be reasonable considering the demand for the product and its observed efficacy, it is high enough that it limits the scale of many metagenomic investigations. For example, studies performing high-throughput analyses on hundreds or thousands of samples may be forced to utilize nonmetagenomic approaches (e.g., 16S rRNA gene sequencing) due to the library preparation expense. In an effort to circumvent this challenge, several alternative and competitive genomic library preparation methods have recently been developed and applied to metagenomic investigations. These approaches fall into two categories: methods that increase the economy of Illumina Nextera by modifying various aspects of the manufacturing protocols (e.g., see reference 21) and those that use entirely different technologies (e.g., seqWell plexWell96). These approaches hold great promise to improve the throughput of metagenomic investigations by reducing library preparation costs. For example, the recent method known as “Hackflex” achieves an 11-fold decrease in per-sample reagent costs compared to the Illumina kit protocols (22).

Although several alternative library preparation approaches have been assessed from the perspective of whole-genome sequencing, very little is known about their accuracy and precision when applied to metagenomic investigations. It is crucial that the performance of novel library preparation procedures be specifically assessed in diverse metagenomic communities as different community types provide unique sequencing challenges not common to traditional whole-genome sequencing. For example, metagenomic communities vary in complexity, with some communities having few distinct taxa (e.g., insect gut) and others being very highly diverse (e.g., soil). Library preparation procedures may vary in their abilities to unbiasedly sample DNA across the different genomes present in the community, whether due to amplification bias in regions with extreme GC content, kit-specific library tagmentation strategies (e.g., enzymatic versus tagmentation), or biases specific to bacterial species present in a community (20). Biological samples vary in their biomass, which affects the amount of whole-community DNA that is subject to the library preparation approach. The sensitivity of these approaches to the amount of input DNA may hence impact study outcomes (23–26).

To advance the utility of low-cost metagenomic library preparation methods, we quantified the performances of five recently developed approaches. Our investigation assessed how different features of metagenomic samples, including community complexity and biomass, impact the performance of these procedures. In particular, we compared Illumina Nextera DNA Flex, a modified DNA Flex protocol (21), Qiagen QIASeq FX DNA, PerkinElmer NextFlex Rapid DNA-Seq 2.0, and seqWell plexWell96 library preparation methods using community-acquired DNA obtained from three different types of microbial communities: low-complexity communities (represented by Acropora hyacinthus microbiomes), moderately complex communities (represented by Mus musculus fecal microbiomes), and a highly complex community (represented by a soil microbiome). We also evaluated how each approach performs on a commercially available mock community comprised of 10 microbial species. Our analysis clarifies the performances of these approaches across these different sample conditions, and the results will assist investigators in identifying appropriate approaches for their metagenomic investigations.

## RESULTS

### Library preparation procedure, community type, and input concentration influence metagenomic library characteristics.

To determine how metagenomic library characteristics (e.g., insert sizes and millions of sequences generated) varied across different metagenomic library preparations, we regressed each library characteristic on community type, library preparation, and input DNA concentration (Table 1). We found that these predictor variables statistically affected the following characteristics: median fragment size [*F*_(31,48)_ = 29.94; *R*^2^ = 0.90; *P < *2.2 × 10^−16^], library concentration [*F*_(31,48)_ = 12.44; *R*^2^ = 0.82; *P* = 3.51 × 10^−14^], library molarity [*F*_(31,48)_ =11.75; *R*^2^ = 0.81; *P *= 1.09 × 10^−13^], sequence read length [*F*_(39,60)_= 56.81; *R*^2^ = 0.96; *P < *2.2 × 10^−16^], number of reads generated [*F*_(39,60)_ = 11.69; *R*^2^ = 0.81; *P < *2.2 × 10^−16^], read GC content [*F*_(39,60)_ = 2,285; *R*^2^ = 0.99; *P < *2.2 × 10^−16^], duplication rate [*F*_(8,91)_ = 2.494; *R*^2^ = 0.11; *P *= 0.02], and percentage of reads filtered [*F*_(39,60)_ = 1,716; *R*^2^ = 0.99; *P < *2.2 × 10^−16^]. The sequence duplication rate was sensitive only to community type [*F*_(3,91)_ = 5.93; *P *= 9.0 × 10^−04^]. Specifically, communities with low microbial diversity such as the coral (*t* = 2.885; *P *= 4.88 × 10^−3^) and mock (*t* = 2.16; *P *= 0.03) communities had elevated duplication rates. All other library characteristics were sensitive to interactions between community type, library preparation, and input DNA concentration, and many characteristics were also impacted by the independent effects of these variables. For example, the library preparation method [*F*_(3,48)_ = 148.85; *P *= 2.61 × 10^−24^] and community type [*F*_(3,48)_ = 11.83; *P *= 6.39 × 10^−06^] affected the median fragment size independent of the interaction between these parameters and the DNA input [*F*_(9,48)_ = 5.93; *P *= 1.56 × 10^−05^]. Library concentration was impacted by community type [*F*_(3,48)_ = 5.72; *P *= 1.98^−03^], library preparation [*F*_(3,48)_ = 20.75; *P *= 9.22 × 10^−09^], input DNA concentration [*F*_(1,48)_ = 186.86; *P < *2.2 × 10^−16^], and their interaction [*F*_(9,48)_ = 6.09; *P *= 1.16 × 10^−05^]. Library molarity was similarly impacted by community type [*F*_(3,48)_ = 5.63; *P *= 2.18 × 10^−03^], library preparation [*F*_(3,48)_ = 35.69; *P *= 2.82 × 10^−12^], input DNA concentration [*F*_(1,48)_ = 126.21; *P *= 4.89 × 10^−15^], and their interaction [*F*_(9,48)_ = 4.40; *P *= 3.16 × 10^−04^].

**TABLE 1:** Library preparation summary statistics^*a*^

Sample type and kit	Median fragment size (bp) (range)	Mean value (range)						
		Library concn (ng/μl)	Library molarity (nM)	Million reads	% reads filtered	Read length (bp)	% duplicates	% GC content
Coral	511.62 (381–670)	12.9 (0.89–35.6)	41.93 (2.11–123.96)	0.75 (0.15–1.43)	68.72 (61.78–74.4)	142.94 (135.02–148.75)	6.03 (3.6–36.12)	38.69 (38–40.33)
Nextera Flex full	616 (542–670)	6.73 (0.89–11.1)	17.41 (2.11–31.51)	1 (0.55–1.43)	69.78 (67.11–72.83)	146.42 (143.75–147.82)	4.91 (4.32–5.3)	38.2 (38–39)
Nextera Flex reduced	527.6 (451–656)	16.69 (6.64–35.6)	46.81 (19.13–83.49)	1 (0.76–1.36)	72.81 (70.49–74.4)	137.46 (135.02–140.24)	3.92 (3.82–4.04)	38 (38–38)
NextFlex Rapid XP	514.6 (443–565)	6.49 (4.09–10)	20.04 (11.14–32.75)	0.66 (0.56–0.78)	64.05 (62.47–66.94)	148.01 (146.94–148.75)	4.7 (4.33–4.97)	38.8 (38–39)
plexWell96	428 (428–428)	9.73 (9.73–9.73)	34.97 (34.97–34.97)	0.23 (0.15–0.37)	71.3 (70.84–71.7)	141.29 (140.26–142.4)	6.43 (5.2–9.48)	40.03 (39.67–40.33)
QIASeq FX	405 (381–417)	22.33 (9.95–33.6)	84.88 (36.71–123.96)	0.83 (0.43–1.16)	65.64 (61.78–68.24)	141.52 (138.03–144.17)	10.17 (3.6–36.12)	38.4 (38–39)

Feces	500.9 (393–652)	12.31 (3.95–53)	40.62 (9.55–207.48)	2.23 (0.39–4.7)	17.76 (12.58–26.88)	141.25 (128.86–148.78)	3.26 (2.17–5.86)	45.36 (43–46)
Nextera Flex full	614.8 (518–652)	9.18 (3.95–24.3)	22.87 (9.55–60.01)	2.83 (1.89–4.7)	17.24 (16.17–18.14)	145.69 (142.76–147.31)	4.99 (4.18–5.86)	45 (45–45)
Nextera Flex reduced	520 (497–548)	12.63 (6.23–26.3)	36.96 (18.22–73.83)	2.64 (1.95–4.16)	26.05 (23.92–26.88)	130.05 (128.86–132.08)	2.58 (2.31–2.95)	43.8 (43–44)
NextFlex Rapid XP	483 (410–529)	9.09 (5.32–20.4)	30.69 (16.67–76.55)	2.64 (2.2–3.4)	16.42 (14.46–17.19)	147.99 (146.2–148.78)	3.03 (2.46–3.37)	46 (46–46)
plexWell96	428 (428–428)	9.73 (9.73–9.73)	34.97 (34.97–34.97)	0.52 (0.39–0.73)	16.29 (15.94–16.59)	140.41 (140.18–140.58)	3.26 (3.1–3.34)	46 (46–46)
QIASeq FX	400.4 (393–409)	18.84 (7.21–53)	73.11 (27.25–207.48)	2.53 (1.8–2.88)	12.79 (12.58–13.13)	142.09 (141.1–143.21)	2.44 (2.17–2.66)	46 (46–46)

Mock	562.86 (388–914)	9.99 (2.8–43.5)	30.21 (7.89–144.86)	3.01 (0.41–6.8)	13.28 (9.03–17.99)	144.26 (138.67–149.18)	5.34 (3.19–8.67)	45.88 (43–48)
Nextera Flex full	625.2 (494–914)	4.38 (2.8–7.3)	10.67 (7.89–14.61)	4.08 (2.22–6.8)	12.24 (11.51–13.92)	145.71 (138.67–147.91)	7.53 (6.37–8.67)	45.4 (45–46)
Nextera Flex reduced	704.2 (640–822)	8.63 (3.58–22.5)	17.84 (8.5–42.11)	3.6 (2.76–4.27)	17.85 (17.73–17.99)	140.63 (139.9–141.49)	6.49 (5.77–7.25)	43 (43–43)
NextFlex Rapid XP	544.2 (462–581)	15.82 (7.86–43.5)	48.47 (20.88–144.86)	3.71 (3.05–4.41)	14.79 (11.35–16.44)	148.87 (148.42–149.18)	4.63 (4.13–5.13)	47.8 (47–48)
plexWell96	428 (428–428)	9.73 (9.73–9.73)	34.97 (34.97–34.97)	0.85 (0.41–1.76)	12.28 (11.77–12.52)	142.31 (140.84–143.63)	4.34 (3.19–5.56)	46.8 (46.33–47)
QIASeq FX	404.8 (388–422)	11.19 (4.5–23.9)	42.89 (17.01–94.77)	2.83 (2.53–3.15)	9.26 (9.03–9.39)	143.81 (143.11–144.77)	3.69 (3.45–4.02)	46.4 (46–47)

Soil	524.33 (375–774)	15.54 (1.83–41.8)	51.55 (5.16–171.49)	2.8 (0.53–5.12)	13.59 (9.05–22.54)	144.45 (138.63–148.5)	2.55 (1.68–3.67)	59.4 (57–61)
Nextera Flex full	515 (443–600)	13.04 (1.83–32.6)	37.68 (5.16–83.59)	2.87 (2.27–3.91)	11.55 (10.63–12.26)	147.36 (145.34–148.5)	2.63 (2.16–3.08)	59 (59–59)
Nextera Flex reduced	743.8 (692–774)	10.41 (5.33–18.4)	21.3 (11.85–36.57)	3.45 (2.03–4.41)	19.74 (17.59–22.54)	140.48 (138.63–142.02)	2.52 (2.31–2.83)	57 (57–57)
NextFlex Rapid XP	468.2 (412–504)	15.48 (7.39–30.2)	52.91 (22.56–112.77)	3.57 (2.75–4.32)	13.38 (11.03–14.26)	147.81 (146.91–148.4)	2.51 (2.08–2.79)	61 (61–61)
plexWell96	428 (428–428)	9.73 (9.73–9.73)	34.97 (34.97–34.97)	0.84 (0.53–1.11)	13.77 (13.14–14.39)	143.25 (142.06–143.83)	3.23 (2.75–3.67)	60 (60–60)
QIASeq FX	389.6 (375–407)	24.38 (12–41.8)	97.62 (45.36–171.49)	3.28 (1.34–5.12)	9.52 (9.05–10.06)	143.36 (142.94–143.65)	1.87 (1.68–1.98)	60 (60–60)

a Each column shows the mean values (ranges) of next-generation sequencing (NGS) library parameters in coral, fecal, mock, and soil sample types.

The number of sequences generated was sensitive to the preparation procedure [*F*_(4,60)_ = 41.68; *P *= 1.10 × 10^−16^], community type [*F*_(3,60)_ = 67.67; *P *= 3.09 × 10^−19^], and DNA input concentration [*F*_(1,60)_ = 5.62; *P *= 2.09 × 10^−02^] as well as the interaction between these variables [*F*_(12,60)_ = 2.25; *P *= 2.00 × 10^−02^]. The number of sequence reads that were quality filtered and derived from the host genome was also significantly affected by this interaction [*F*_(12,60)_ = 5.16; *P *= 7.44 × 10^−06^]. Metagenome libraries constructed for coral communities had increased levels of quality filtering (*t* = 62.11; *P *= 3.66 × 10^−56^), while filtering in soil (*t* = −6.65; *P *= 9.84 × 10^−09^) and mock (*t* = −6.56; *P *= 1.39^−08^) communities was decreased compared to fecal samples. Read filtering was also consistently increased in samples prepared with the Nextera Flex reduced method (*t* = −12.38; *P *= 3.59^−18^) and reduced in the samples prepared using the QIASeq FX procedure (*t* = −4.89; *P *= 7.84^−06^).

Metagenomic read characteristics were also significantly impacted by the examined variables. For example, the GC content of reads was significantly dependent on the community type [*F*_(3,60)_ = 2.89 × 10^4^; *P *= 9.38 × 10^−95^]. GC content was also affected by the library preparation method [*F*_(4,60)_ = 442.80; *P *= 8.71 × 10^−44^]; a significant effect of the interaction between library preparation, community type, and input DNA concentration [*F*_(12,60)_ = 2.68; *P *= 5.98 × 10^−03^] was also observed for GC content. Finally, average read length after quality filtering varied by both community type [*F*_(3,60)_ = 60.78; *P *= 3.54 × 10^−18^] and preparation procedure [*F*_(4,60)_ = 404.25; *P *= 1.21 × 10^−42^]. Collectively, these findings indicate that metagenomic library preparation procedures yield distinct library characteristics for different community types.

### Different library preparation methods result in similar taxonomic profiles of a standardized mock community.

**FIG 1:**
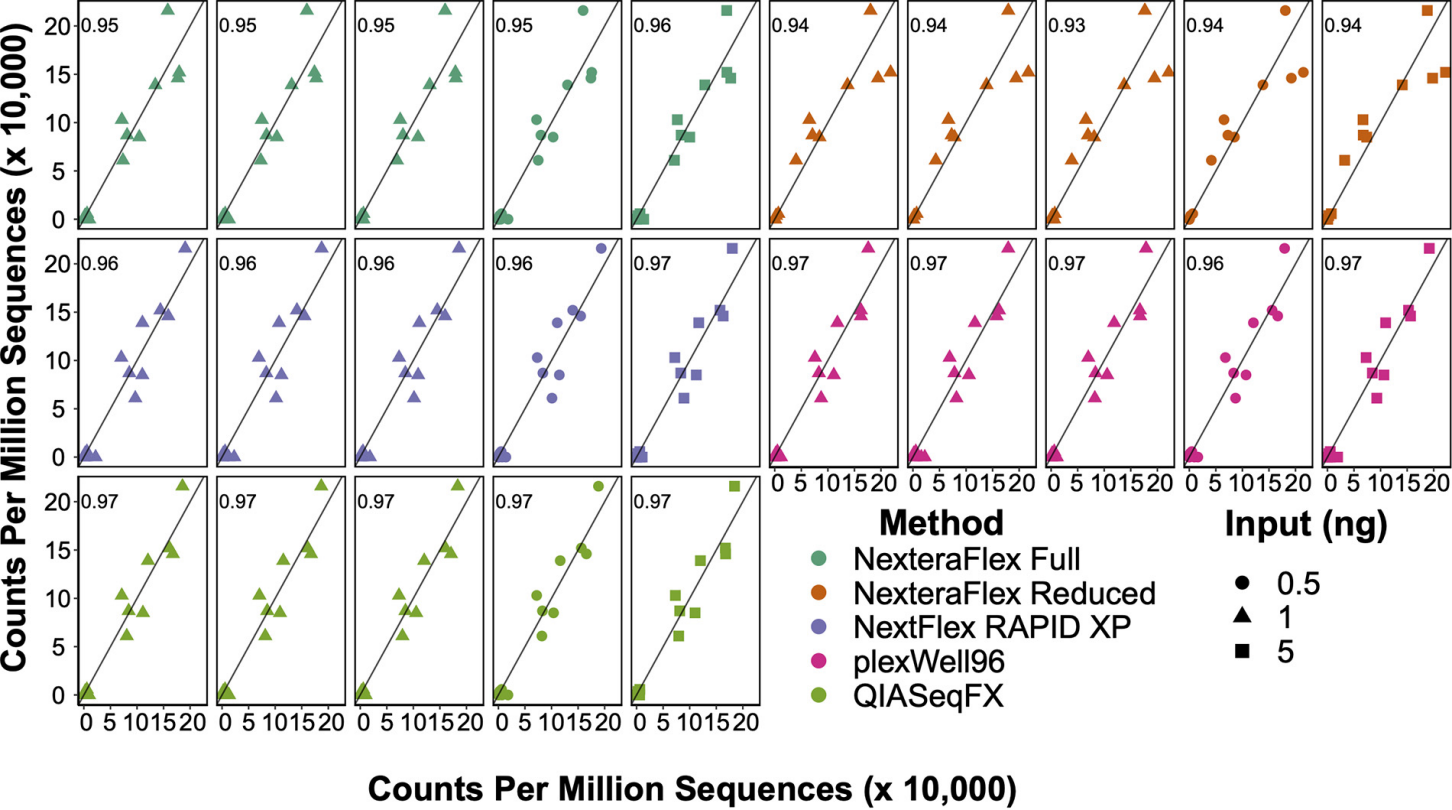
Metagenomic library preparation methods accurately predict the taxonomic composition of a simplified mock community. Scatterplots of the observed and theoretical taxonomic compositions of a mock community are shown. The library preparation methodology is indicated by point colors, and the input concentration is denoted by point shapes. Values in each panel represent the Pearson correlation coefficients.

While library preparation procedures vary in the resulting metagenomic library and sequence characteristics, it is unclear if this variation results in different downstream assessments of community composition. To address this question, we quantified how each library preparation method predicted the taxonomic composition of a defined mock community. We compared the taxonomic composition generated by each library preparation to the ZymoBIOMICS microbial community standard’s defined taxonomic composition of the mock community. Strong correlations (ρ = 0.93 to 0.97; *P *= 1.29 × 10^−6^ to *P < *2.2 × 10^−16^; FDR [false discovery rate] < 1.0 × 10^−5^) were observed between the MetaPhlAn2-inferred taxonomic abundances and the theoretical taxonomic abundances of taxa present in the mock community (Fig. 1; see also Table S1 in the supplemental material). To confirm that this was not due to bias in the MetaPhlAn2 database, we also compared the inferred taxonomic abundances using Kraken2 and observed similar taxonomic associations (ρ = 0.93 to 0.96; all *P < *2.2 × 10^−16^; FDR < 2.2 × 10^−16^) and abundance profiles (Fig. S1).

10.1128/mSystems.00440-21.1 FIG S1 Kraken2-imputed abundance profiles strongly correlate with theoretical mock community compositions across all metagenomic library preparation methods. Scatterplots of the inferred Kraken2 taxonomic profiles and theoretical taxonomic compositions of a mock community are shown. The library preparation methodology is indicated by point colors, and the input concentration is denoted by point shapes. Values in each panel represent the Pearson correlation coefficients. Download FIG S1, EPS file, 0.4 MB. Copyright © 2021 Gaulke et al. 2021 Gaulke et al. https://creativecommons.org/licenses/by/4.0/ This content is distributed under the terms of the Creative Commons Attribution 4.0 International license.

10.1128/mSystems.00440-21.2 TABLE S1 Observed mock community abundances are highly similar to theoretical abundances. Observed and theoretical taxon abundances for mock community samples prepared with five library preparation procedures at three input concentrations are shown. Fold differences between theoretical counts per million (CPM) and observed CPM were modest. Download Table S1, CSV file, 0.02 MB. Copyright © 2021 Gaulke et al. 2021 Gaulke et al. https://creativecommons.org/licenses/by/4.0/ This content is distributed under the terms of the Creative Commons Attribution 4.0 International license.

The MetaPhlAn2 results showed that the Nextera Flex full and Nextera Flex reduced methods, which are widely used in metagenomic studies, had the lowest correlations (ρ = 0.93 to 0.96; *P *= 1.29 × 10^−6^ to 2.66 × 10^−8^) with the theoretical composition of the mock community. The lower correlations produced by these two library preparation procedures are driven by an underestimation of the abundance of Lactobacillus fermentum and an overestimation of the abundances of Staphylococcus aureus and Enterococcus faecalis. The strongest correlations were observed with the QIASeq FX (ρ = 0.97; *P *= 1.21 × 10^−8^ to 5.79 × 10^−9^), plexWell96 (ρ = 0.96 to 0.97; *P *= 2.58 × 10^−8^ to 1.24 × 10^−8^), and NextFlex Rapid (ρ = 0.96 to 0.97; *P *= 1.02 × 10^−7^ to 2.38 × 10^−8^) methods (Fig. 1). Together, these data indicate that library preparation methods subtly influence some taxonomic estimates but that all methods examined overall performed well at recapitulating simple, defined microbial communities.

### Community taxonomic profiles are significantly impacted by library preparation procedure and input concentration.

**FIG 2:**
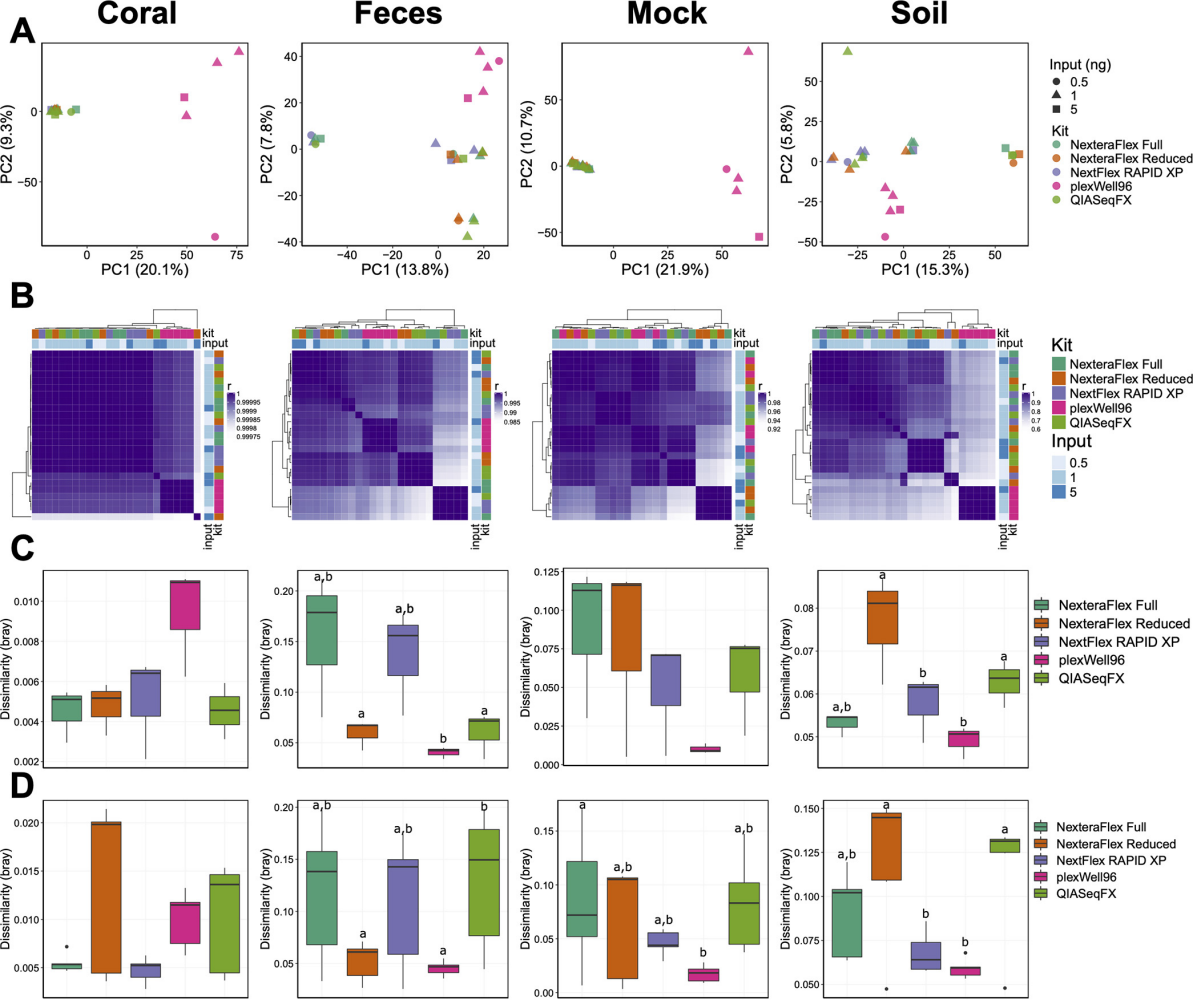
Microbial taxonomic diversity varies by library preparation method and input concentration. (A) PCA ordinations of microbial taxonomic diversity (Kraken2) for each sample type. (B) Correlation heat maps of taxonomic abundances generated from each library type. For each preparation method, a 0.5-ng input (*n* = 1), a 1.0-ng input (*n* = 3), and a 5-ng input (*n* = 1) were used. Row and column side plots indicate the library preparation methodology and the input concentration. (C and D) Box plots of dissimilarities (Bray-Curtis) among technical replicates (*n* = 3 [1-ng inputs]) (C) and different input concentrations, 0.5 ng (*n* = 1), 1.0 ng (*n* = 3), and 5 ng (*n* = 1) (D). Letters indicate significant differences (*P < *0.05).

Next, we quantified the impact of library preparation methods and input concentrations on the species-level taxonomic profiles of soil, coral, mock, and fecal metagenomes. The library preparation procedure was significantly associated with the resulting taxonomic microbiome profiles as measured by permutational multivariate analysis of variance (PERMANOVA) in coral (*R*^2^ = 0.87; *P *= 2.00 × 10^−4^) and soil (*R*^2^ = 0.33; *P *= 5.00 × 10^−3^) but not in fecal (*R*^2^ = 0.32; *P *= 0.06) or mock (*R*^2^ = 0.26; *P *= 0.15) communities (Fig. 2A). In the fecal and mock communities, no association was found between the input concentration and microbiome beta-diversity. However, in coral (*R*^2^ = 0.02; *P *= 0.02) and soil (*R*^2^ = 0.13; *P *= 5.4 × 10^−3^) communities, we identified a significant association between diversity and input. We also identified a significant interaction effect between input concentration and metagenomic preparation method in coral (*R*^2^ = 0.07; *P *= 1.4 × 10^−3^). The taxonomic abundance profiles of each library were highly correlated (ρ = 0.63 to 1; FDR < 2.2 × 10^−16^) across library preparation methods (Fig. 2B), suggesting that the preparation methodology associates with distinct community profiles and offering a high level of profile prediction precision between each preparation method.

To quantify how variable individual replicates were across different library preparation methods, we examined the dissimilarity in taxonomic beta-diversity within each library preparation method. Methods that yield low intraconcentration (i.e., 1-ng/μl input) dissimilarities indicate high levels of reproducibility, while methods with low interconcentration dissimilarities (i.e., all concentrations) would indicate that the taxonomic profiles generated using this method are robust to variation in the library input concentration. We found that variation in intraconcentration taxonomic dissimilarity was low, and modest significant differences were observed only in fecal communities (*H* = 10.2; *P *= 0.04) across library preparation methodologies (Fig. 2C). The interconcentration dissimilarity was also low in all communities but varied significantly in fecal (*H* = 12.3; *P *= 0.02), mock (*H* = 11.6; *P *= 0.02), and soil (*H* = 15.6; *P *= 3.6 × 10^−3^) samples but across preparation methods potentially due to variance in dissimilarity for the Nextera Flex reduced and plexWell96 libraries (Fig. 2D). Together, these data suggest that the taxonomic profiles generated using the methods under investigation are similarly reproducible, but the robustness varied across methods.

### Community gene abundance profiles are sensitive to library preparation procedures and input DNA concentrations.

**FIG 3:**
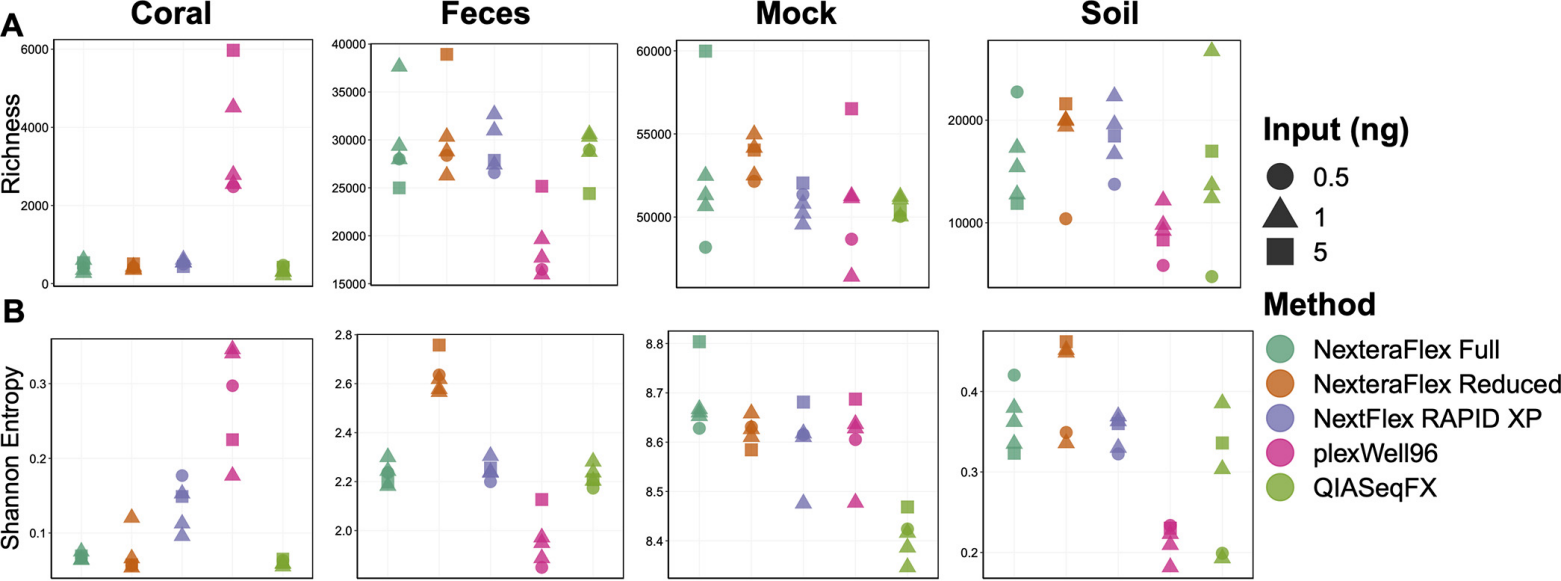
Metagenomic diversity varies by library preparation method and input concentration. Gene richness (A) and Shannon entropy (B) plots for each sample type are shown.

Metagenomic investigations frequently seek to define the genetic diversity of microbial communities. Using the number of distinct gene families observed in the data (i.e., gene family richness) as well as the functional composition of the community (i.e., gene family beta-diversity), we measured how different library preparation procedures affected the determination of a community’s functional capacity. Gene family richness varied by library preparation method and input concentration in coral [*F*_(9,15)_ = 31.90; *R*^2^ = 0.92; *P *= 3.47 × 10^−08^], fecal [*F*_(9,15)_ = 9.13; *R*^2^ = 0.75; *P *= 1.20 × 10^−04^], and mock [*F*_(9,15)_ = 8.78; *R*^2^ = 0.74; *P *= 1.51 × 10^−04^] communities, while soil richness [*F*_(9,15)_ = 1.41; *R*^2^ = 0.13; *P* = 0.27] was less sensitive to these effects (Fig. 3A). We also observed a significant interaction between library preparation procedure and DNA input on the predicted functional profiles of coral [*F*_(4,15)_ = 8.00; *P *= 1.16 × 10^−03^], fecal [*F*_(4,15)_ = 5.64; *P *= 5.60 × 10^−03^], and mock [*F*_(4,15)_ = 7.78; *P *= 1.33 × 10^−03^] microbiomes. However, these associations were not always consistent across different library preparation procedures. For example, gene richness was elevated in coral samples prepared with the plexWell96 method (*t* = 5.53; *P *= 5.77 × 10^−05^), while a contrasting pattern was observed in both fecal (*t* = −6.38; *P *= 1.23 × 10^−05^) and soil (*t* = −1.958; *P *= 6.91 × 10^−02^) samples, and no difference was identified in mock community samples (*t* = −0.58; *P *= 0.57). Similar effects of library preparation method and input concentration were observed on Shannon entropy (Fig. 3B). Specifically, significant effects were observed for coral [*F*_(9,15)_ = 11.09; *R*^2^ = 0.79; *P *= 3.75 × 10^−05^], fecal [*F*_(9,15)_ = 62.29; *R*^2^ = 0.96; *P *= 1.89 × 10^−10^], mock [*F*_(9,15)_ = 11.04; *R*^2^ = 0.79; *P *= 3.85 × 10^−05^], and soil [*F*_(9,15)_ = 5.06; *R*^2^ = 0.60; *P *= 2.95 × 10^−03^] samples.

**FIG 4:**
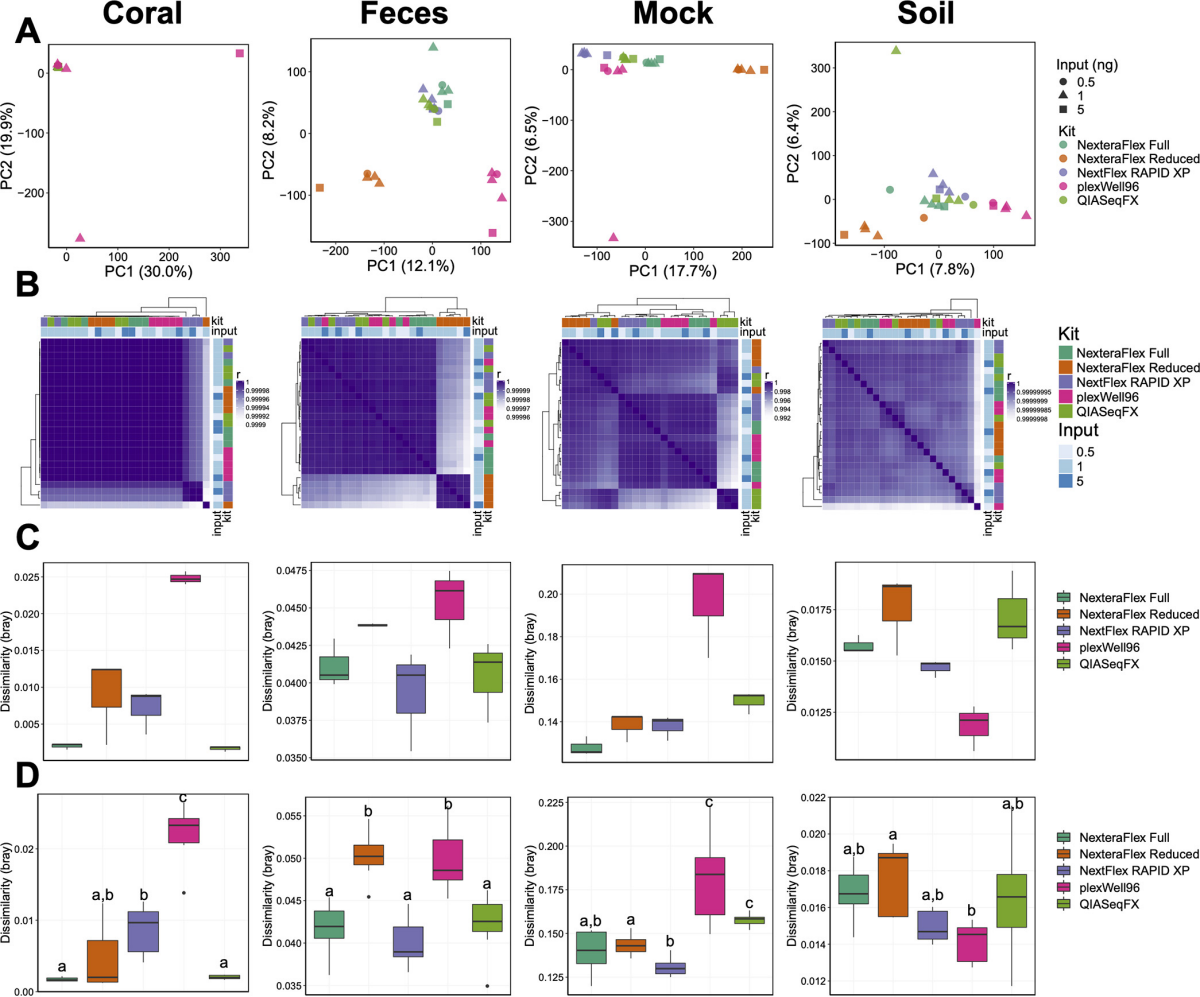
Microbial functional diversity varies by library preparation method and input concentration. (A) PCA ordinations of gene family beta-diversity for each sample type. (B) Correlation heat maps of gene family abundances generated from each library type. Row and column side plots indicate the library preparation methodology and the input concentration. For each preparation method, a 0.5-ng input (*n* = 1), a 1.0-ng input (*n* = 3), and a 5-ng input (*n* = 1) were used. (C and D) Box plots of dissimilarities (Bray-Curtis) among technical replicates (*n* = 3 [1-ng inputs]) (C) and different input concentrations, 0.5 ng (*n* = 1), 1.0 ng (*n* = 3), and 5 ng (*n *= 1) (D). Letters indicate significant differences (*P < *0.05).

As measured by PERMANOVA, we found that gene family beta-diversity (Bray-Curtis) was significantly associated with library preparation method for coral [*F*_(4,15)_ = 3.62; *R*^2^ = 0.44; *P *= 2.00 × 10^−04^], fecal [*F*_(4,15)_ = 7.82; *R*^2^ = 0.60; *P *= 2.00 × 10^−04^], mock [*F*_(4,15)_ = 3.36; *R*^2^ = 0.41; *P *= 2.00 × 10^−04^], and soil [*F*_(4,15)_ = 3.39; *R*^2^ = 0.40; *P *= 2.00 × 10^−04^] communities (Fig. 4A). However, the association between library preparation procedure and gene family beta-diversity is muted in comparison to taxonomic beta-diversity (Fig. 2A), possibly as a result of the increased overall similarity in gene family abundances across samples of the same community type (ρ = 0.99 to 1.00; *P < *2.2 × 10^−16^) (Fig. 4B). Despite this increased similarity between library preparation methods for gene family abundances, the beta-diversity of technical replicates varied across library preparation methods for coral (*H* = 11.43; *P *= 2.21 × 10^−02^), fecal (*H* = 9.03; *P *= 6.02 × 10^−02^), mock (*H* = 12.1; *P *= 1.66 × 10^−02^), and soil (*H* = 11.5; *P *= 2.15 × 10^−02^) samples. However, significant differences were not detected between individual library preparation methods (Fig. 4C). The robustness of metagenome beta-diversity to input concentration differed across library preparation methods for coral (*H* = 24.33; *P *= 6.86 × 10^−05^), fecal (*H* = 24.53; *P *= 6.26 × 10^−05^), mock (*H* = 25.65; *P *= 3.72 × 10^−05^), and soil (*H* = 13.83; *P *= 7.85 × 10^−03^) samples (Fig. 4D). Notably, the plexWell96 method had elevated variability compared to the other library prep methods in coral, fecal, and mock community samples. In soil, these patterns were mitigated (Fig. 4D). Overall, these data demonstrate that, similar to the taxonomic profiles, library preparation methods affect gene diversity profile predictions.

## DISCUSSION

### Taxonomic and functional profile predictions are similar across methodologies.

**FIG 5:**
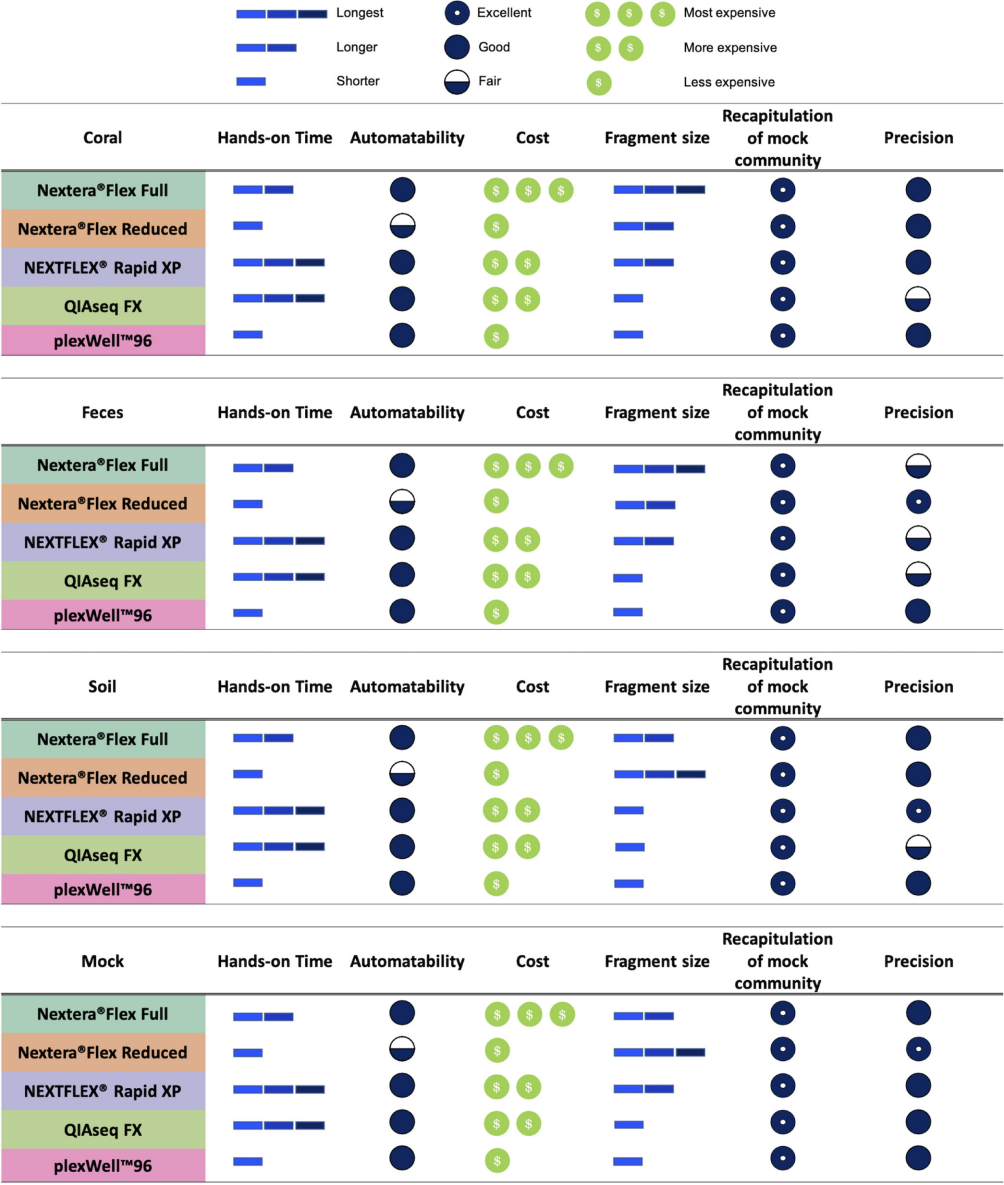
Library preparation summary and cost metric reference guide. Hands-on time refers to the active time necessary for essential benchwork tasks. Fragment size categories are relative to other kit fragment sizes for each sample type. Recapitulation of mock community refers to the correlation coefficient of a given mock community and the community produced by each kit. Precision refers to the level of variability in taxonomic community composition between 1.0-ng DNA input technical replicates for each sample type.

Although the Nextera kits are widely used and considered the gold standard for metagenomic sample preparation, their cost can limit researchers from conducting expansive project aims. As applications for metagenomic sequencing continue to increase, researchers are left with the difficult task of balancing the need for high-quality data with the cost of their generation. The development of new protocols that modify the standard Nextera kit protocol as well as several new economical library preparation kits has the potential to dramatically alter the field by expanding the accessibility of shotgun metagenomics. However, the quality of libraries prepared using more economical methods varies substantially (19). While previous studies have demonstrated that different library preparation procedures can affect metagenome characteristics (27–30), these studies did not evaluate contemporary procedures, nor did they consider the sensitivity of the approaches to different metagenome sample types. Here, we demonstrate that library quality as well as taxonomic and functional profiles vary as a function of environmental community type and biomass. Our findings suggest that while researchers need to be aware of differences between kits, overall, the taxonomic and functional profiles produced are similar and grant comparable precision among the kits (Fig. 5).

Several investigations have identified key differences in library characteristics across metagenomic library preparation procedures, often by incorporating multiple study designs. These variations can result in substantial changes in the quality of the metagenomic library and are important considerations in preparation method selection. For example, Baym et al. demonstrated that a custom Nextera XT protocol yielded a substantially reduced insert fragment length (21). Smaller fragments have higher proportions of adapter contamination in reads, while fragments that are too large may be preferentially lost during the Illumina cluster generation process (31). We observed significant effects of community type, library preparation procedure, and input DNA concentration on fragment size. In our hands, the Nextera Flex protocols generated the largest library insert sizes, while the QIASeq FX and plexWell96 procedures consistently produced the smallest. However, the Nextera procedures also produced libraries with the lowest average GC content compared to the other procedures examined. This reduced representation of GC content could impact the representation of genes with high GC content and skew both taxonomic and functional profiles (19, 32). The interacting effects of library preparation procedure, community type, and DNA input on GC content further indicate that specific library preparation procedures may have distinct insertion site biases.

Comparing library characteristics across environmental sample types, samples with low relative diversity (i.e., coral) had both a high percentage of duplicate reads and a high number of reads filtered and removed from the resulting libraries regardless of the input concentration. This high level of filtering is likely due to the extreme levels of host DNA contaminants relative to the other sample types and additionally may point to the larger issue of host sequence contamination, regardless of the library preparation method, in similar research studies. However, coral samples also had similar levels of precision across kit types, with the exception of lower precision with QIASeq FX, demonstrating that different kit types may still be viable options in other low-complexity study systems.

Samples with moderate (fecal) and high (soil) microbial diversity had much lower respective average percentages of sequencing reads filtered than coral samples but with a higher average GC content across libraries than coral samples. Fecal sample libraries had the highest variability in precision between kit types, with the exception of Nextera Flex reduced, likely due to the more complex community composition. However, our study also had a relatively low average sequencing depth across all samples due to sample number and financial constraints; the high intracommunity variability in precision that we observed may be resolved with higher sequencing coverage (33). It is also possible that longer insert fragment sizes introduce greater variability due to lower base quality in the produced community composition regardless of sample type (34), although this was not the case for coral, soil, or mock libraries with the longest fragment sizes.

In successfully recapitulating the taxonomic profiles of a mock microbial community, all library preparation methods performed similarly overall; however, variation in taxonomic profiles for the environmental sample types showed subtle differences between methods. While higher levels of intracommunity variation per method could again be due to low sequencing depth, our results of higher variation for the coral sample types with lower relative diversity are consistent with previous findings that library coverage is increased for highly complex microbial communities. Furthermore, while it may appear that all preparation methods perform poorly in both taxonomic and functional resolution for low (coral)- and high (soil)-diversity sample types, it must be noted that these profiles may only be as complete as the reference databases used for assignment, and it is well known that these databases are preferentially curated with human microbiome sequences and studies in mind (35).

### Financial and opportunity costs of metagenomic preparation methods differ.

Decreasing the costs of kits and reagents associated with library preparation improves access to metagenomic approaches. The Nextera DNA Flex full preparation actualized cost remains the most expensive of the five methods tested, with NextFlex Rapid XP and QIASeq FX in the median relative expense range and Nextera Flex reduced prep and plexWell96 as the most economical choices for metagenomic library generation. However, due to the above-noted effect of the specificity of the environmental sample type on the performance of the preparation method, neither the most economical choice nor the most expensive may necessarily suit every study or generate the highest-quality libraries. Due to the effects of preparation procedure, community type, and DNA input on fragment size and both taxonomic and functional profiles of metagenomic samples, comparing communities across multiple study designs may require additional covariates in statistical design. For future studies, we recommend incorporating the library preparation technique as a potential covariate in statistical design to account for these known differences and potential biases.

Finally, one important limitation of our study is the lack of biological variation (i.e., only one sample per community type), which makes it challenging to determine whether technical variation inherently matters to a particular study. For some applications, such as biomarker discovery, technical variance may contribute to decreased sensitivity and specificity if technical biases exist for a library preparation procedure. On the other hand, technical variation is expected to have more modest impacts on studies of differential gene abundance across case and control groups because biological variance will likely overwhelm technical variance. Thus, the interpretation of the impact of the technical variation on future analyses should be carefully considered within the context of the biological variation of their specific application.

### Conclusion.

Collectively, these findings demonstrate that no single metagenomic library preparation approach performed the best across all community types and conditions evaluated. Rather, the performance of approaches varied as a function of the sample and the amount of input DNA. Consequently, researchers should consider these variables when selecting library preparation approaches, especially when attempting to optimize data quality, accuracy, and precision. To aid in this effort, we provide Fig. 5 as a reference guide to aid in choosing preparation methods with cost and performance in mind. We hope that this information helps improve the accessibility and utility of metagenomic investigations. Further study is needed to determine what community properties (e.g., GC content and taxonomic diversity, etc.) dictate these differences in library procedure performance in order to generate more generalizable guidance for procedure selection. That said, our results show that the different approaches generally produced relatively consistent taxonomic and gene family diversity profiles, which indicates that selecting approaches based on cost and ease of implementation may be appropriate for some studies (namely, those in which the loss of accuracy and precision is tolerable). However, we recommend careful consideration of the community type and the amount of input DNA when selecting a metagenomic library preparation procedure to ensure optimal performance.

## MATERIALS AND METHODS

### Genomic DNA extraction.

Prior to metagenomic library construction, genomic DNA was extracted from environmental samples originating from soil from the North American Project To Evaluate Soil Health Measurements (36), coral (*Acropora hyacinthus*), and mammalian feces (Mus musculus; C57BL/6), using methods outlined below. In addition to environmental samples, we used the ZymoBIOMICS microbial community DNA standard (catalog number D6306, lot number ZRC193008) to more efficiently assess bias associated with library preparation methods on a standard mock community.

For the coral slurry, coral nubbins preserved in RNA/DNA Shield (ZymoBIOMICS) were vortexed in 15-ml tubes with a combination of ceramic and garnet bead lysing matrices at ∼2,500 rpm for 25 min. DNA was extracted from 300 μl of the resulting coral slurry using the ZymoBIOMICS DNA/RNA miniprep kit (Zymo Research Corp., Irvine, CA, USA) following an additional 2-step enzyme incubation to increase the recovery of bacterial DNA: (i) the addition of 30 μl chicken egg white lysozyme (10 mg/ml; Novagen), 1.8 μl mutanolysin (50,000 units/ml from Streptomyces globisporus ATCC 21553; Sigma-Aldrich), and 1.8 μl lysostaphin (4 KU/ml from Staphylococcus *staphylolyticus*; Sigma-Aldrich) with incubation at 37°C for 1 h and (ii) 1 h of incubation at 50°C following the addition of 15 μl proteinase K (20 mg/ml; Thermo Scientific) and 30 μl proteinase K digestion buffer (0.1 M NaCl, 10 mM Tris [pH 9.0], 1 mM EDTA, 0.5% SDS, nuclease-free water). Following digestion, 1 volume of kit-specific DNA/RNA lysis buffer was added in order to proceed with the manufacturer’s recommended extraction protocol.

For soil, the sample was taken on 27 February 2019 at the Virginia Tech Eastern Shore Agricultural Research and Extension Center. Samples were collected as 12 composite knife slices of soil to a depth of 15 cm, and each of the 12 slices was passed through an 8-mm filter. Detailed sampling methods were described previously by Norris et al. (36). Following collection, 0.25-g aliquots of soil were stored at −80°C after overnight shipment from the collection site. Soil aliquots were then extracted according to the Earth Microbiome Project protocol (37) using a KingFisher Flex kit (Thermo Fisher).

For mouse feces, DNA was isolated from a single fecal pellet using the DNeasy PowerSoil isolation kit (Qiagen) according to the manufacturer’s instructions. An additional 10-min incubation step at 65°C directly before bead beating was added to enhance microbial cell lysis. The samples were then homogenized using Vortex-Genie 2 and a vortex adapter (Qiagen) at the highest setting for 10 min.

### Metagenomic library preparation and sequencing.

Environmental DNA samples were prepared for metagenomic sequencing according to the manufacturers’ protocols using the following four commercially available kits: (i) the Illumina Nextera DNA Flex library kit, (ii) the Qiagen QIASeq FX DNA library kit, (iii) PerkinElmer NextFlex Rapid DNA-Seq kit 2.0, and (iv) seqWell plexWell96. In addition, we included a fifth preparation method using the modified “reduced” protocol established by Baym et al. to increase the number of libraries that Nextera DNA Flex could generate (21). Genomic DNA was quantified using a Qubit 1× high-sensitivity (HS) double-stranded DNA (dsDNA) assay kit for soil, fecal, and coral communities. The mock community DNA concentration was not quantified as ZymoBIOMICS manufacturer information provided a known concentration of 100 ng/μl. Following quantification, all samples prepared using Nextera Flex full, QIASeq FX, and NextFlex Rapid XP were diluted with water to 0.2 ng/μl. To determine how the DNA input affected library generation, each standardized DNA concentration was then added to obtain the respective 0.5-ng, 1.0-ng, and 5.0-ng inputs. Samples prepared using plexWell96 were diluted to 0.25 ng/μl with water, and appropriate additive volumes were made to obtain 0.5-ng and 1.0-ng input concentrations. For samples with 5.0-ng inputs, samples were diluted to 1.25 ng/μl, and 4 μl of the sample was then used to obtain a 5.0-ng input concentration. For the Nextera Flex reduced reaction, all samples were diluted to 5.0 ng/μl with nuclease-free water. A 1-μl aliquot of this dilution was used for 5.0-ng input libraries. For 0.5-ng and 1.0-ng input libraries, sufficient water was added to the 1-μl dilution to bring the respective concentrations to 0.5 ng/μl and 1.0 ng/μl.

The library insert size was assessed for the Nextera DNA Flex (full and reduced) and plexWell96 methods using Agilent TapeStation 4200 high-sensitivity D5000 DNA ScreenTape. The insert size for the QIASeq FX and NextFlex Rapid XP methods was quantified using the Agilent Bioanalyzer 2100 high-sensitivity DNA chip as these libraries are more prone to having adapter dimers, which are poorly resolved using the TapeStation. The library concentration was assessed with the Qubit 1× high-sensitivity dsDNA quantification kit (Thermo Fisher). The resulting libraries were normalized to the lowest concentration for each library prep kit based on molarity using the Qubit concentration and Bioanalyzer/TapeStation median fragment sizes (except for plexWell96, which pools early on in the library prep procedure). All 20 of the normalized samples from each kit were then pooled, and each of the 5 pools was verified using quantitative PCR (qPCR). These 5 pools were normalized and pooled just prior to sequencing for paired-end reads of 150 bp on a single lane with the Illumina HiSeq3000 system.

### Microbial community gene family abundance and taxonomic diversity.

Quality filtering, adapter removal, and host read filtering were performed using shotcleaner v0.1 (38) with default parameters. For mouse fecal samples, host reads were removed by alignment to the mouse reference genome (GRCm38). A similar procedure was used for coral samples except that these reads were filtered against a concatenated version of the coral (Acropora millepora; GenBank accession number QTZP00000000.1 [39]) and symbiont (*Symbiodiniaceae* sp. clade A MAC-Cass KB8 [UniProt taxon identifier 671378]) genomes. Quality-controlled sequence reads were input into HUMAnN 2.0 (40) for taxonomic and functional classification using the UniRef90 database and default parameters. HUMAnN 2.0 outputs for each community type were combined and renormalized to counts per million using HUMAnN 2.0 utility scripts before downstream analysis. High-quality reads were also taxonomically classified using Kraken2 v2.0.8-beta (41, 42) and a custom reference database that included sequences from all human, mouse (GRCm38), UniVec core, bacterial, archaeal, viral, fungal, and protozoal sequences in the NCBI RefSeq database (accessed 8 October 2019) as well as the *Symbiodiniaceae* sp. clade A MAC-Cass KB8 and A. millepora genomes. Taxonomic data derived from Kraken2 were normalized by dividing the number of taxonomic annotations within a given hierarchy by the total number of overall reads annotated (i.e., relative abundances of different bacterial species, genera, or phyla).

### Statistical analyses.

Independent linear models (R::stats::lm) were used to determine how community type, library preparation method, and input DNA concentration affect the variance of the resulting library characteristics, including the number of reads generated, median fragment size, library concentration, library molarity, mean read length, read duplication rate, mean read GC content, and total reads filtered and removed. Since we reasoned that it was likely that interactions between the predictors exist, we employed a model selection procedure to identify the most parsimonious model for each characteristic examined. For each characteristic, we built a set of models of increasing complexity: (i) a reduced model with only additive effects (equation 1) and (ii) a model with interaction terms for community type, library preparation procedure, and DNA input concentration (equation 2).

(1) $$\text{Characteristic }=\beta_{0}\;+\;\beta_{\text{1}}\left(\text{community}\right)\;+\;\beta_{\text{2}}\left(\text{library preparation}\right)\;+\;\beta_{\text{3}}\left(\text{DNA input}\right)\;+\;\varepsilon$$

(2) $$\text{Characteristic }=\beta_{0}\;+\;\beta_{\text{1}}\left(\text{community}\right)\;\times \;\beta_{\text{2}}\left(\text{library preparation}\right)\;\times \;\beta_{\text{3}}\left(\text{DNA input}\right)\;+\varepsilon$$

We then used the Akaike information criterion (AIC) to select the most parsimonious model and analysis of variance (ANOVA) to determine the significance of each term in the selected model [*F*(*df*_1_ = *K* − 1, *df*_2_ = *n* − *K*, α = 0.05)]. Because sequencing libraries produced from distinct samples are pooled as part of the plexWell library preparation protocol, a single value is available for median fragment size, sequence library concentration, and sequence library molarity for this method.

The similarity between taxonomic profiles generated by the library preparation methods and the known taxonomic composition of the ZymoBIOMICS microbial community DNA standard was assessed by Pearson’s correlation test (R::stats::cor.test). To measure the variation in species-level taxonomic profiles generated by different library preparation methods across soil, coral, and fecal communities, we calculated Pearson’s correlation coefficient of the generated taxonomic abundance profiles for each pair of samples. This analysis was conducted using Kraken2, a sensitive read-binning tool, and MetaPhlAn2, a marker-gene-based abundance estimation tool to eliminate the possibility that mammalian biases in marker gene databases would skew results in environmental samples. We accounted for the effects of multiple correlation tests using the false discovery rate (R::stats::p.adjust, method = fdr).

The additive and interactive statistical effects of library preparation and DNA input concentration on the microbiome composition, as measured by the Bray-Curtis dissimilarity metric, were evaluated using PERMANOVA (R::vegan::adonis, permutations = 5,000, method= bray) and visualized using an ordination of principal-component analysis (PCA) for each community. Differences in the Bray-Curtis dissimilarity of species-level taxonomic abundance profiles within and across library preparation methods were measured using Kruskal-Wallis tests (R::stats::kruskal.test) with a *post hoc* pairwise Wilcoxon test (R::stats::pairwise.wilcox.test). A Holm correction was used to control Wilcoxon test family-wise error rates.

Shannon entropy and gene richness were calculated for HUMAnN 2.0 gene abundance profiles using R and vegan. Linear regression quantified associations between gene-level alpha-diversity and library preparation and input DNA concentration for each community type. Associations with gene-level Bray-Curtis dissimilarity, preparation method, and DNA input were quantified using PERMANOVA (R::vegan::adonis, permutations = 5,000, method = bray). Differences in gene abundances and metagenomic dissimilarity were quantified as described above for taxonomy.

### Data availability.

Raw sequence data are available from the NCBI under BioProject accession number PRJNA747032. The GitHub code repository for reference of processing and analyzing raw data is available at https://github.com/chrisgaulke/ht_metagenomes.

## References

[1] Paszkiewicz K, Studholme DJ. 2010. De novo assembly of short sequence reads. Brief Bioinform 11:457–472. doi:10.1093/bib/bbq020. 20724458

[2] Brumfield KD, Huq A, Colwell RR, Olds JL, Leddy MB. 2020. Microbial resolution of whole genome shotgun and 16S amplicon metagenomic sequencing using publicly available NEON data. PLoS One 15:e0228899. doi:10.1371/journal.pone.0228899. 32053657 PMC7018008

[3] Yoon SS, Kim E-K, Lee W-J. 2015. Functional genomic and metagenomic approaches to understanding gut microbiota-animal mutualism. Curr Opin Microbiol 24:38–46. doi:10.1016/j.mib.2015.01.007. 25625313

[4] Quince C, Walker AW, Simpson JT, Loman NJ, Segata N. 2017. Shotgun metagenomics, from sampling to analysis. Nat Biotechnol 35:833–844. doi:10.1038/nbt.3935. 28898207

[5] Laudadio I, Fulci V, Palone F, Stronati L, Cucchiara S, Carissimi C. 2018. Quantitative assessment of shotgun metagenomics and 16S rDNA amplicon sequencing in the study of human gut microbiome. OMICS 22:248–254. doi:10.1089/omi.2018.0013. 29652573

[6] Jovel J, Patterson J, Wang W, Hotte N, O’Keefe S, Mitchel T, Perry T, Kao D, Mason AL, Madsen KL, Wong GK-S. 2016. Characterization of the gut microbiome using 16S or shotgun metagenomics. Front Microbiol 7:459. doi:10.3389/fmicb.2016.00459. 27148170 PMC4837688

[7] Human Microbiome Project Consortium. 2012. Structure, function and diversity of the healthy human microbiome. Nature 486:207–214. doi:10.1038/nature11234. 22699609 PMC3564958

[8] Armour CR, Nayfach S, Pollard KS, Sharpton TJ. 2019. A metagenomic meta-analysis reveals functional signatures of health and disease in the human gut microbiome. mSystems 4:e00332-18. doi:10.1128/mSystems.00332-18. 31098399 PMC6517693

[9] Guo J, Li J, Chen H, Bond PL, Yuan Z. 2017. Metagenomic analysis reveals wastewater treatment plants as hotspots of antibiotic resistance genes and mobile genetic elements. Water Res 123:468–478. doi:10.1016/j.watres.2017.07.002. 28689130

[10] Jadeja NB, Purohit HJ, Kapley A. 2019. Decoding microbial community intelligence through metagenomics for efficient wastewater treatment. Funct Integr Genomics 19:839–851. doi:10.1007/s10142-019-00681-4. 31111267

[11] Li A-D, Li L-G, Zhang T. 2015. Exploring antibiotic resistance genes and metal resistance genes in plasmid metagenomes from wastewater treatment plants. Front Microbiol 6:1025. doi:10.3389/fmicb.2015.01025. 26441947 PMC4585309

[12] Wang Z, Zhang X-X, Huang K, Miao Y, Shi P, Liu B, Long C, Li A. 2013. Metagenomic profiling of antibiotic resistance genes and mobile genetic elements in a tannery wastewater treatment plant. PLoS One 8:e76079. doi:10.1371/journal.pone.0076079. 24098424 PMC3787945

[13] Fierer N, Lauber CL, Ramirez KS, Zaneveld J, Bradford MA, Knight R. 2012. Comparative metagenomic, phylogenetic and physiological analyses of soil microbial communities across nitrogen gradients. ISME J 6:1007–1017. doi:10.1038/ismej.2011.159. 22134642 PMC3329107

[14] Blaya J, Marhuenda FC, Pascual JA, Ros M. 2016. Microbiota characterization of compost using omics approaches opens new perspectives for Phytophthora root rot control. PLoS One 11:e0158048. doi:10.1371/journal.pone.0158048. 27490955 PMC4973912

[15] Lutz S, Thuerig B, Oberhaensli T, Mayerhofer J, Fuchs JG, Widmer F, Freimoser FM, Ahrens CH. 2020. Harnessing the microbiomes of suppressive composts for plant protection: from metagenomes to beneficial microorganisms and reliable diagnostics. Front Microbiol 11:1810. doi:10.3389/fmicb.2020.01810. 32849417 PMC7406687

[16] Blockley A, Elliott DR, Roberts AP, Sweet M. 2017. Symbiotic microbes from marine invertebrates: driving a new era of natural product drug discovery. Diversity 9:49. doi:10.3390/d9040049.

[17] Trindade M, van Zyl LJ, Navarro-Fernández J, Abd Elrazak A. 2015. Targeted metagenomics as a tool to tap into marine natural product diversity for the discovery and production of drug candidates. Front Microbiol 6:890. doi:10.3389/fmicb.2015.00890. 26379658 PMC4552006

[18] Kennedy J, Marchesi JR, Dobson ADW. 2007. Metagenomic approaches to exploit the biotechnological potential of the microbial consortia of marine sponges. Appl Microbiol Biotechnol 75:11–20. doi:10.1007/s00253-007-0875-2. 17318533

[19] Jones MB, Highlander SK, Anderson EL, Li W, Dayrit M, Klitgord N, Fabani MM, Seguritan V, Green J, Pride DT, Yooseph S, Biggs W, Nelson KE, Venter JC. 2015. Library preparation methodology can influence genomic and functional predictions in human microbiome research. Proc Natl Acad Sci USA 112:14024–14029. doi:10.1073/pnas.1519288112. 26512100 PMC4653211

[20] Sato MP, Ogura Y, Nakamura K, Nishida R, Gotoh Y, Hayashi M, Hisatsune J, Sugai M, Takehiko I, Hayashi T. 2019. Comparison of the sequencing bias of currently available library preparation kits for Illumina sequencing of bacterial genomes and metagenomes. DNA Res 26:391–398. doi:10.1093/dnares/dsz017. 31364694 PMC6796507

[21] Baym M, Kryazhimskiy S, Lieberman TD, Chung H, Desai MM, Kishony R. 2015. Inexpensive multiplexed library preparation for megabase-sized genomes. PLoS One 10:e0128036. doi:10.1371/journal.pone.0128036. 26000737 PMC4441430

[22] Gaio D, To J, Liu M, Monahan L, Anantanawat K, Darling AE. 2019. Hackflex: low cost Illumina sequencing library construction for high sample counts. bioRxiv 779215. 10.1099/mgen.0.000744 PMC8914357 35014949

[23] Marine R, Polson SW, Ravel J, Hatfull G, Russell D, Sullivan M, Syed F, Dumas M, Wommack KE. 2011. Evaluation of a transposase protocol for rapid generation of shotgun high-throughput sequencing libraries from nanogram quantities of DNA. Appl Environ Microbiol 77:8071–8079. doi:10.1128/AEM.05610-11. 21948828 PMC3209006

[24] Solonenko SA, Ignacio-Espinoza JC, Alberti A, Cruaud C, Hallam S, Konstantinidis K, Tyson G, Wincker P, Sullivan MB. 2013. Sequencing platform and library preparation choices impact viral metagenomes. BMC Genomics 14:320. doi:10.1186/1471-2164-14-320. 23663384 PMC3655917

[25] Chafee M, Maignien L, Simmons SL. 2015. The effects of variable sample biomass on comparative metagenomics. Environ Microbiol 17:2239–2253. doi:10.1111/1462-2920.12668. 25329041

[26] Duhaime MB, Deng L, Poulos BT, Sullivan MB. 2012. Towards quantitative metagenomics of wild viruses and other ultra-low concentration DNA samples: a rigorous assessment and optimization of the linker amplification method. Environ Microbiol 14:2526–2537. doi:10.1111/j.1462-2920.2012.02791.x. 22713159 PMC3466414

[27] Mandal S, Treuren WV, White RA, Eggesbø M, Knight R, Peddada SD. 2015. Analysis of composition of microbiomes: a novel method for studying microbial composition. Microb Ecol Health Dis 26:27663. doi:10.3402/mehd.v26.27663. 26028277 PMC4450248

[28] Simon C, Daniel R. 2011. Metagenomic analyses: past and future trends. Appl Environ Microbiol 77:1153–1161. doi:10.1128/AEM.02345-10. 21169428 PMC3067235

[29] Pawlowsky-Glahn V, Egozcue JJ, Tolosana-Delgado R. 2015. Modeling and analysis of compositional data. John Wiley & Sons, Chichester, United Kingdom.

[30] Gloor GB, Macklaim JM, Pawlowsky-Glahn V, Egozcue JJ. 2017. Microbiome datasets are compositional: and this is not optional. Front Microbiol 8:2224. doi:10.3389/fmicb.2017.02224. 29187837 PMC5695134

[31] Kircher M, Heyn P, Kelso J. 2011. Addressing challenges in the production and analysis of Illumina sequencing data. BMC Genomics 12:382. doi:10.1186/1471-2164-12-382. 21801405 PMC3163567

[32] Browne PD, Nielsen TK, Kot W, Aggerholm A, Gilbert MTP, Puetz L, Rasmussen M, Zervas A, Hansen LH. 2020. GC bias affects genomic and metagenomic reconstructions, underrepresenting GC-poor organisms. Gigascience 9:giaa008. doi:10.1093/gigascience/giaa008. 32052832 PMC7016772

[33] Rodriguez-R LM, Konstantinidis KT. 2014. Estimating coverage in metagenomic data sets and why it matters. ISME J 8:2349–2351. doi:10.1038/ismej.2014.76. 24824669 PMC4992084

[34] Tan G, Opitz L, Schlapbach R, Rehrauer H. 2019. Long fragments achieve lower base quality in Illumina paired-end sequencing. Sci Rep 9:2856. doi:10.1038/s41598-019-39076-7. 30814542 PMC6393434

[35] Lindgreen S, Adair KL, Gardner PP. 2016. An evaluation of the accuracy and speed of metagenome analysis tools. Sci Rep 6:19233. doi:10.1038/srep19233. 26778510 PMC4726098

[36] Norris CE, Bean GM, Cappellazzi SB, Cope M, Greub KLH, Liptzin D, Rieke EL, Tracy PW, Morgan CLS, Honeycutt CW. 2020. Introducing the North American project to evaluate soil health measurements. Agron J 112:3195–3215. doi:10.1002/agj2.20234.

[37] Thompson LR, Sanders JG, McDonald D, Amir A, Ladau J, Locey KJ, Prill RJ, Tripathi A, Gibbons SM, Ackermann G, Navas-Molina JA, Janssen S, Kopylova E, Vázquez-Baeza Y, González A, Morton JT, Mirarab S, Zech Xu Z, Jiang L, Haroon MF, Kanbar J, Zhu Q, Jin Song S, Kosciolek T, Bokulich NA, Lefler J, Brislawn CJ, Humphrey G, Owens SM, Hampton-Marcell J, Berg-Lyons D, McKenzie V, Fierer N, Fuhrman JA, Clauset A, Stevens RL, Shade A, Pollard KS, Goodwin KD, Jansson JK, Gilbert JA, Knight R, Earth Microbiome Project Consortium. 2017. A communal catalogue reveals Earth’s multiscale microbial diversity. Nature 551:457–463. doi:10.1038/nature24621. 29088705 PMC6192678

[38] Sharpton T. 2017. sharpton/shotcleaner. Perl6.

[39] Ying H, Hayward DC, Cooke I, Wang W, Moya A, Siemering KR, Sprungala S, Ball EE, Forêt S, Miller DJ. 2019. The whole-genome sequence of the coral Acropora millepora. Genome Biol Evol 11:1374–1379. doi:10.1093/gbe/evz077. 31059562 PMC6501875

[40] Franzosa EA, McIver LJ, Rahnavard G, Thompson LR, Schirmer M, Weingart G, Lipson KS, Knight R, Caporaso JG, Segata N, Huttenhower C. 2018. Species-level functional profiling of metagenomes and metatranscriptomes. Nat Methods 15:962–968. doi:10.1038/s41592-018-0176-y. 30377376 PMC6235447

[41] Wood DE, Salzberg SL. 2014. Kraken: ultrafast metagenomic sequence classification using exact alignments. Genome Biol 15:R46. doi:10.1186/gb-2014-15-3-r46. 24580807 PMC4053813

[42] Wood DE, Lu J, Langmead B. 2019. Improved metagenomic analysis with Kraken 2. Genome Biol 20:257. doi:10.1186/s13059-019-1891-0. 31779668 PMC6883579

